# Expression and gene regulation network of *RBM8A* in hepatocellular carcinoma based on data mining

**DOI:** 10.18632/aging.101749

**Published:** 2019-01-22

**Authors:** Yan Lin, Rong Liang, Yufen Qiu, Yufeng Lv, Jinyan Zhang, Gang Qin, Chunling Yuan, Zhihui Liu, Yongqiang Li, Donghua Zou, Yingwei Mao

**Affiliations:** ^1^ Department of Medical Oncology, Affiliated Tumor Hospital of Guangxi Medical University, Nanning, Guangxi 530021, People’s Republic of China; ^2^ Maternal and Child Health Hospital and Obstetrics and Gynecology Hospital of Guangxi Zhuang Autonomous Region, Guangxi 530021, People’s Republic of China; ^3^ Department of Medical Oncology, Affiliated Langdong Hospital of Guangxi Medical University, Nanning, Guangxi 530021, People’s Republic of China; ^4^ The Fifth Affiliated Hospital of Guangxi Medical University, Nanning, Guangxi 530021, People’s Republic of China; ^5^ Department of Biology, Pennsylvania State University, University Park, PA 16802, USA; ^*^ Equal contribution

**Keywords:** *RBM8A*, HCC, prognosis, functional network analysis

## Abstract

RNA binding motif protein 8A (RBM8A) is an RNA binding protein in a core component of the exon junction complex. Abnormal *RBM8A* expression is associated with carcinogenesis. We used sequencing data from the Cancer Genome Atlas database and Gene Expression Omnibus, analyzed *RBM8A* expression and gene regulation networks in hepatocellular carcinoma (HCC). Expression was analyzed using Oncomine^TM^ and Gene Expression Profiling Interactive Analysis tools, while *RBM8A* alterations and related functional networks were identified using cBioPortal. LinkedOmics was used to identify differential gene expression with *RBM8A* and to analyze Gene Ontology and Kyoto Encyclopedia of Genes and Genomes pathways. Gene enrichment analysis examined target networks of kinases, miRNAs and transcription factors. We found that *RBM8A* is overexpressed and the *RBM8A* gene often amplified in HCC. Expression of this gene is linked to functional networks involving the ribosome and RNA metabolic signaling pathways. Functional network analysis suggested that *RBM8A* regulates the spliceosome, ribosome, DNA replication and cell cycle signaling via pathways involving several cancer-related kinases, miRNAs and E2F Transcription Factor 1. Our results demonstrate that data mining efficiently reveals information about *RBM8A* expression and potential regulatory networks in HCC, laying a foundation for further study of the role of *RBM8A* in carcinogenesis.

## INTRODUCTION

Hepatocellular carcinoma (HCC) is one of the top ten malignant tumors and the third leading cause of cancer-related death in the world [1]. China accounts for 55% of new HCC cases and HCC-related deaths every year [2]. Due to the high recurrence and metastasis, the 5-year survival rate of patients with advanced HCC does not exceed 5% [3]. The development of various targeted drugs have prolonged the survival of patients and made a revolutionary breakthrough in the treatment of advanced HCC. However, existing targeted drugs show unsatisfactory efficacy. Even with sorafenib or regorafenib therapy, the overall life expectancy of HCC patients is less than 1 year [4, 5]. The pathogenesis of HCC is extremely complex, involving processes such as cell cycle regulation and signal transduction, which reflects the function and interaction of multiple genes at multiple steps [6]. It may be possible to identify new drug targets for HCC by screening gene networks for changes related to tumor formation and progression.

RNA binding motif protein 8A (RBM8A), also known as Y14, is a conserved protein widely expressed in cells, and is an RNA binding motif protein [7]. RBM8A serves as a core factor of the RNA surveillance machinery for the exon junction complex (EJC). It is also known to be the core protein of nonsense-mediated mRNA decay (NMD), which monitors abnormal mRNA in eukaryotes [8, 9]. RBM8A is related to RNA transcription, translation, cell cycle regulation and apoptosis regulation [10, 11], and it is also involved in several crucial cell signaling pathways [12–15], in which it plays an important role in tumorigenesis and development. Abnormal expression of RBM8A was first detected in cervical cancer [7], and its overexpression was subsequently detected in various malignant tumors, including non-small cell lung cancer and myeloma [16–18].

In our studies with patient samples from Guangxi, one of the regions with the highest incidence of HCC in China, we found that RBM8A was overexpressed in HCC tumor tissues compared to normal liver tissues, and this overexpression was associated with the surface antigen of the hepatitis B virus (HBsAg) and Edmondson pathological grading. Moreover, Kaplan-Meier survival analysis showed that high RBM8A expression was associated with poor overall survival and progression-free survival of HCC patients. Gain- and loss-of-function experiments further demonstrated that RBM8A promotes invasion and metastasis via the signaling pathway involved in the endothelial-to-mesenchymal (EMT) transition, while loss of RBM8A induces apoptosis [19].

These results suggest that RBM8A is a novel proto-oncogene. Thus, we studied RBM8A expression and mutations in data from patients with HCC in The Cancer Genome Atlas (TCGA) and various public databases. Using multi-dimensional analysis methods, we analyzed genomic alterations and functional networks related to RBM8A in HCC. Thus, our results could potentially reveal new targets and strategies for HCC diagnosis and treatment.

## RESULTS

### *RBM8A* expression in HCC

**Figure 1:**
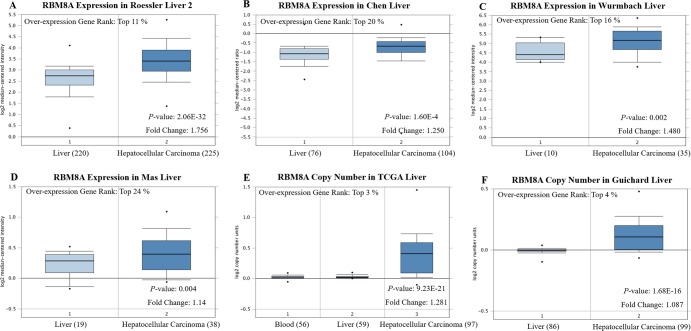
***RBM8A* transcription in hepatocellular carcinoma (Oncomine).** Levels of *RBM8A* mRNA and DNA copy number were significantly higher in hepatocellular carcinoma than in normal tissue. Shown are fold change, associated *p* values, and overexpression gene rank, based on Oncomine 4.5 analysis. (**A–D**) Box plot showing *RBM8A* mRNA levels in, respectively, the Roessler Liver 2, Chen Liver, Wurmbach Liver and Mas Liver datasets. (**E–F**) Box plot showing *RBM8A* copy number in The Cancer Genome Atlas (TCGA) Liver and Guichard Liver datasets, respectively.

**Figure 2:**
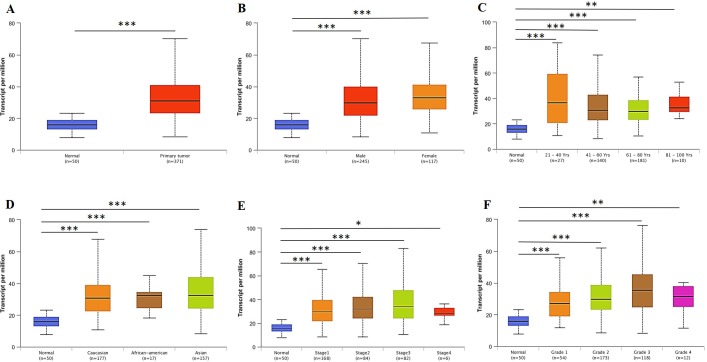
***RBM8A* transcription in subgroups of patients with hepatocellular carcinoma, stratified based on gender, age and other criteria (UALCAN).** (**A**) Boxplot showing relative expression of *RBM8A* in normal and LIHC samples. (**B**) Boxplot showing relative expression of *RBM8A* in normal individuals of either gender or male or female LIHC patients. (**C**) Boxplot showing relative expression of *RBM8A* in normal individuals of any age or in LIHC patients aged 21–40, 41–60, 61–80, or 81–100 yr. (**D**) Boxplot showing relative expression of *RBM8A* in normal individuals of any ethnicity or in LIHC patients of Caucasian, African-American or Asian ethnicity. (**E**) Boxplot showing relative expression of *RBM8A* in normal individuals or in LIHC patients in stages 1, 2, 3 or 4. (**F**) Boxplot showing relative expression of *RBM8A* in normal individuals or LIHC patients with grade 1, 2, 3 or 4 tumors. Data are mean ± SE. *, *P* < 0.05; **, *P* < 0.01; ***, *P* < 0.001.

We initially evaluated *RBM8A* transcription levels in multiple HCC studies from TCGA and the Gene Expression Omnibus (GEO). Data in the Oncomine 4.5 database revealed that mRNA expression and DNA copy number variation (CNV) of *RBM8A* were significantly higher in HCC tissues than in normal tissues (*p* < 0.01). Although the fold differences were within 2, *RBM8A* ranked within the top 25% based on mRNA expression and within the top 5% based on DNA CNVs (Figure 1). Further sub-group analysis of multiple clinic pathological features of 371 liver hepatocellular carcinoma (LIHC) samples in the TCGA consistently showed high transcription of *RBM8A*. The transcription level of *RBM8A* was significantly higher in HCC patients than healthy people in subgroup analyses based on gender, age, ethnicity, disease stages and tumor grade (Figure 2). Thus, *RBM8A* expression may serve as a potential diagnostic indicator in HCC.

### Genomic alterations of *RBM8A* in HCC

### *Frequency and type of RBM8A alterations in HCC*

**Figure 3:**
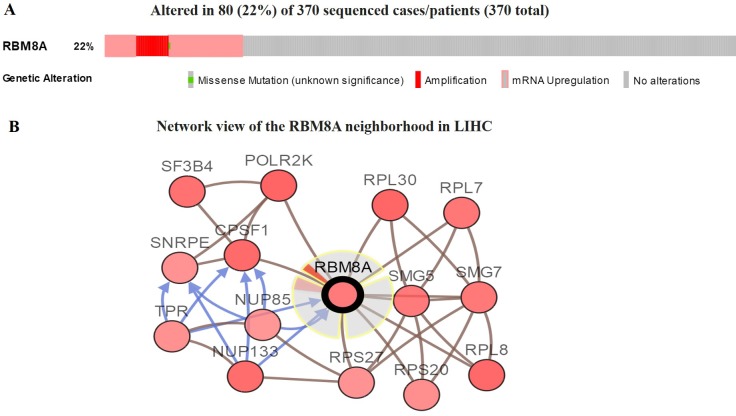
**Visual summary of *RBM8A* alterations and biological interaction network in hepatocellular carcinoma (cBioPortal).** (**A**) OncoPrint of *RBM8A* alterations in LIHC. The OncoPrint provides an overview of genomic alterations in *RBM8A* affecting individual samples (columns) in LIHC from the TCGA. The different types of genetic alterations are highlighted in different colors. (**B**) Network view of the *RBM8A* neighborhood in LIHC. *RBM8A* are seed genes (indicated with thick border), and all other genes are automatically identified as altered in LIHC. Darker red indicates increased frequency of alteration in LIHC. The interaction types are derived from the Biological Pathway Exchange (BioPAX): the blue connection indicates that the first protein controls a reaction that changes the state of the second protein; the red connection indicates that the proteins are members of the same complex.

We then used the cBioPortal to determine the types and frequency of *RBM8A* alterations in HCC based on sequencing data from LIHC patients in the TCGA database. *RBM8A* was altered in 80 of 370 (22%) LIHC patients (Figure 3A). These alterations were mRNA upregulation in 60 cases (16%), amplification in 38 cases (10%), mutation in 1 case (0.3%), and multiple alterations in 19 cases (5%) (Table 1). Thus, amplification is the most common type of *RBM8A* CNV in HCC.

**Table 1:** The type and frequency of RBM8A neighbor gene alterations in hepatocellular carcinoma (cBioPortal)

Gene Symbol	Amplification	Homozygous Deletion	Up-regulation	Down-regulation	Mutation	Total Alteration
RBM8A	10.1	0	16.1	0	0.3	21.6
CPSF1	16.1	0	30.9	0	2.2	37.2
NUP133	8.7	0	29.8	0.8	2.5	35.8
NUP85	5.7	0	17.8	0	0.5	21.0
POLR2K	15.6	0.3	37.7	0.3	0	40.7
RPL30	14.8	0.3	34.7	0	0	38.8
RPL7	11.2	0	28.1	0	0	31.4
RPL8	16.1	0	32.2	0	0.3	37.7
RPS20	7.1	0	19.1	0	0	23.2
RPS27	12.3	0	11.5	0	0.3	22.1
SF3B4	10.7	0	30.3	0	0.5	33.6
SMG5	12.3	0	21.6	0	1.1	30.3
SMG7	9.6	0	27.6	0	0.8	31.1
SNRPE	9.6	0	14.2	0	0	22.4
TPR	8.7	0	16.1	0.3	1.4	22.7

### *Biological interaction network of RBM8A alterations in HCC*

**Figure 4:**
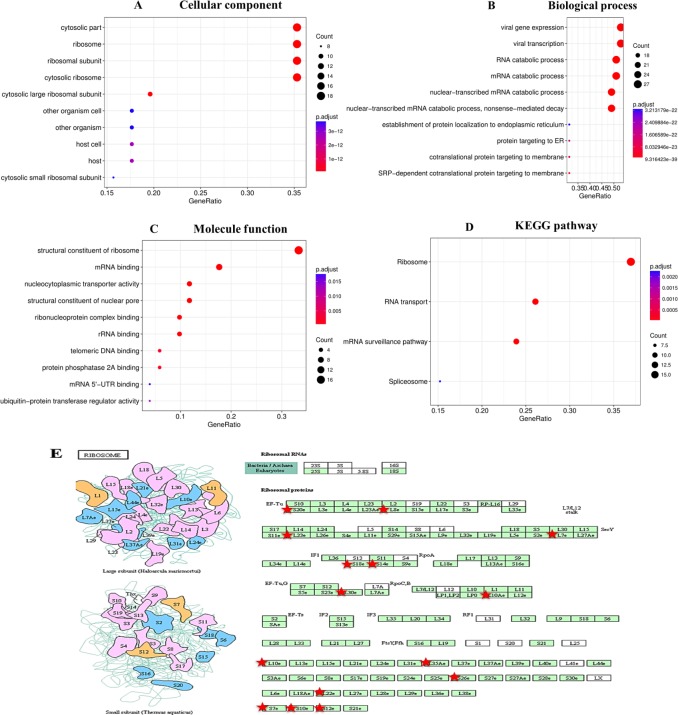
**Enrichment analysis of the genes altered in the *RBM8A* neighborhood in hepatocellular carcinoma.** The bubble diagrams display the enrichment results of the top 50 genes altered in the *RBM8A* neighborhood in LIHC. (**A**) Cellular components. (**B**) Biological processes. (**C**) Molecular functions. (**D**) KEGG pathway analysis. (**E**) KEGG pathway annotations of the ribosome pathway. The red star denotes altered genes.

We next wanted to determine the biological interaction network of *RBM8A* in HCC. To do this, we used the tab *Network* in cBioPortal to show *RBM8A*-neighboring genes that were altered at frequencies >20% (Figure 3B and Table 1). The neighbor genes of *RBM8A* with the most frequent alterations were *POLR2K* (40.7%), *RPL30* (38.8%) and *CPSF1* (37.2%). The 50 most frequently altered neighbor genes were determined using *Network*. Analysis of significantly enriched gene ontology (GO) terms indicated that these genes encode proteins localized mainly to the cytosol, ribosome, and ribosome subunits. These proteins are primarily involved in viral gene expression and RNA catabolism, while they also serve as structural constituents of ribosomes and mRNA binding (Figure 4A–4C). Similarly, Kyoto Encyclopedia of Genes and Genomes (KEGG) pathway analysis showed enrichment in ribosome signaling, RNA transport, mRNA surveillance and spliceosome signaling pathways (Figure 4D, 4E). Thus, the biological interaction network of *RBM8A* alterations is involved in the ribosome and several RNA metabolic processes.

### Enrichment analysis of *RBM8A* functional networks in HCC

### *GO and KEGG pathway analyses of co-expression genes correlated with RBM8A in HCC*

**Figure 5:**
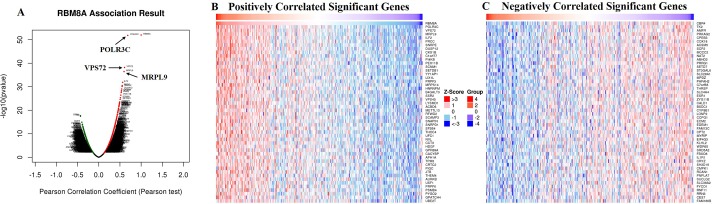
**Genes differentially expressed in correlation with *RBM8A* in hepatocellular carcinoma (LinkedOmics).** (**A**) A Pearson test was used to analyze correlations between *RBM8A* and genes differentially expressed in LIHC. (**B–C**) Heat maps showing genes positively and negatively correlated with *RBM8A* in LIHC (TOP 50). Red indicates positively correlated genes and green indicates negatively correlated genes.

**Figure 6:**
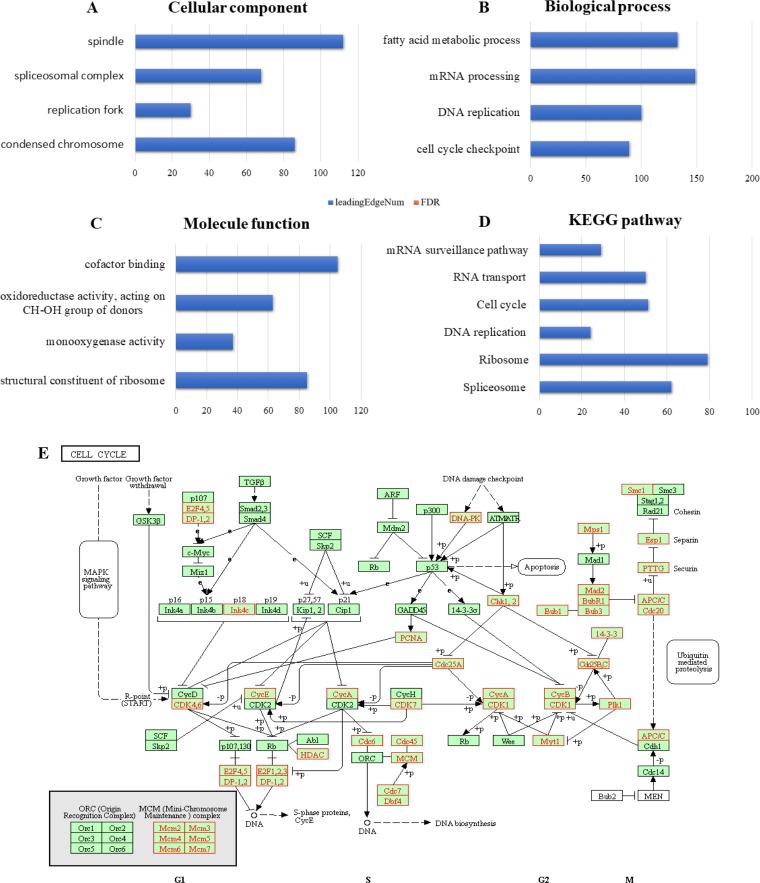
**Significantly enriched GO annotations and KEGG pathways of *RBM8A* in hepatocellular carcinoma.** The significantly enriched GO annotations and KEGG pathways of *RBM8A* co-expression genes in LIHC were analyzed using GSEA. (**A**) Cellular components. (**B**) Biological processes. (**C**) Molecular functions. (**D**) KEGG pathway analysis. The blue column represents the LeadingEdgeNum, and the orange represents the false discovery rate (FDR). The FDR from GSEA in the figure is 0. (**E**) KEGG pathway annotations of the cell cycle pathway. Red marked nodes are associated with the LeadingEdgeGene.

The *Function* module of LinkedOmics was used to analyze mRNA sequencing data from 371 LIHC patients in the TCGA. As shown in the volcano plot (Figure 5A), 2,596 genes (dark red dots) showed significant positive correlations with *RBM8A*, whereas 3,050 genes (dark green dots) showed significant negative correlations (false discovery rate [FDR] < 0.01). The 50 significant gene sets positively and negatively correlated with *RBM8A* as shown in the heat map (Figure 5B, 5C). This result suggests a widespread impact of *RBM8A* on the transcriptome. The statistical scatter plots for individual genes are shown in Supplementary Figure 1A–1C. *RBM8A* expression showed a strong positive association with expression of *POLR3C* (positive rank #1, Pearson correlation = 0.68, *p* = 1.48e–52), *VPS72* (Pearson correlation = 0.61, *p* = 6.64e–39), and *MRPL9* (Pearson correlation = 0.60, *p* = 3.64e–37), which reflect changes in RNA polymerase III transcription initiation, transcriptional regulation/DNA repair/apoptosis and mitochondrial ribosome composition. Significant GO term analysis by gene set enrichment analysis (GSEA) showed that genes differentially expressed in correlation with *RBM8A* were located mainly in the condensed chromosome, replication fork and spliceosome complex, where they participate primarily in cell cycle checkpoint control, DNA replication and mRNA processing. They act as structural constituents in ribosomes and monooxygenase (Figure 6A–6C and Supplementary Tables 1–3). KEGG pathway analysis showed enrichment in the spliceosome, ribosome, DNA replication and cell cycle pathways (Figure 6D, 6E and Supplementary Table 4).

### *RBM8A networks of kinase, miRNA or transcription factor targets in HCC*

**Figure 7:**
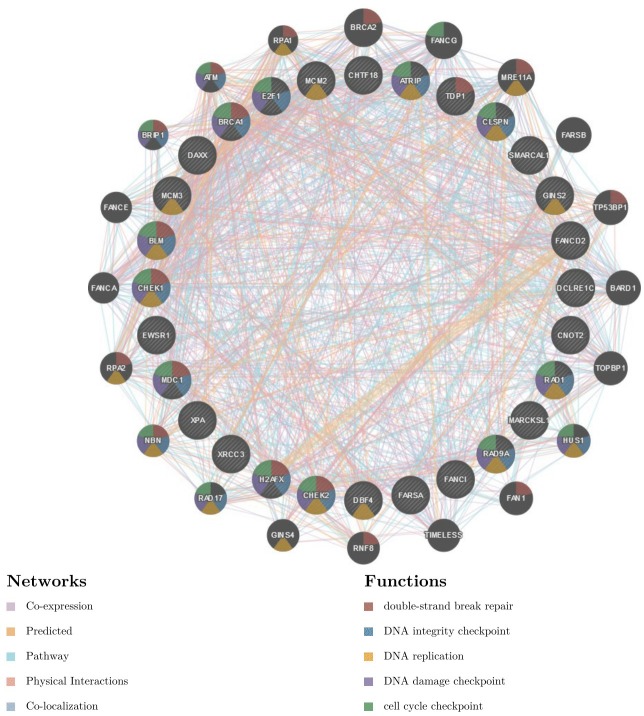
**Protein-protein interaction network of ATR kinase-target networks (GeneMANIA).** Protein-protein interaction (PPI) network and functional analysis indicating the gene set that was enriched in the target network of ATR kinases. Different colors of the network edge indicate the bioinformatics methods applied: co-expression, website prediction, pathway, physical interactions and co-localization. The different colors for the network nodes indicate the biological functions of the set of enrichment genes.

To further explore the targets of *RBM8A* in HCC, we analyzed the kinase, miRNA and transcription factor target networks of positively correlated gene sets generated by GSEA. The top 5 most significant target networks were the kinase-target networks related primarily to the kinases ataxia telangiectasia-mutated and Rad3-related (ATR), Aurora kinase B (AURKB), cyclin-dependent kinase 1 (CDK1), cyclin-dependent kinase 2 (CDK2) and checkpoint kinase 1 (CHEK1) (Table 2 and Supplementary Tables 5–7). The miRNA-target network was associated with (CTTTGCA) MIR-527, (CCCACAT) MIR-299-3P and (GTGCAAT) MIR-25, MIR-32, MIR-92, MIR-363, and MIR-367. The transcription factor-target network was related mainly to the E2F Transcription Factor (E2F) family, including E2F1DP1RB_01, E2F1DP1_01 and E2F1DP2_01. The protein-protein interaction network constructed by GeneMANIA revealed correlation among genes for the kinases ATR, miRNA-527 and TF E2F1DP1RB_01. The gene set enriched for kinase ATR and transcription factor E2F1DP1RB is responsible mainly for regulating DNA replication, DNA repair and the cell cycle checkpoint (Figure 7 and Supplementary Figure 3). In addition, the gene set enriched for transcription factor E2F1DP1RB is also involved in the G1/S transition in mitosis, telomere maintenance and the MCM complex. The gene set enriched for miRNA-527 is involved mainly in regulation of the tricarboxylic acid cycle, organ development and angiogenesis (Supplementary Figure 2).

**Table 2:** The Kinase, miRNA and transcription factor-target networks of RBM8A in in hepatocellular carcinoma (LinkedOmics)

Enriched Category	Geneset	LeadingEdgeNum	FDR
**Kinase Target**	Kinase_ATR	28	0
	Kinase_AURKB	34	0
	Kinase_CDK1	95	0
	Kinase_CDK2	110	0
	Kinase_CHEK1	47	0
**miRNA Target**	CTTTGCA, MIR-527	71	0.025
	CCCACAT, MIR-299-3P	23	0.025
	GTGCAAT, MIR-25, MIR-32, MIR-92, MIR-363, MIR-367	78	0.029
	GACAATC, MIR-219	49	0.030
	ACTTTAT, MIR-142-5P	85	0.030
**Transcription Factor Target**	V$E2F1DP1RB_01	76	0
	V$E2F1DP1_01	80	0
	V$E2F1DP2_01	80	0
	V$E2F1_Q3	76	0
	V$E2F1_Q4_01	82	0

Abbreviations: LeadingEdgeNum, the number of leading edge genes; FDR, false discovery rate from Benjamini and Hochberg from gene set enrichment analysis (GSEA). V$, the annotation found in Molecular Signatures Database (MSigDB) for transcription factors (TF).

## DISCUSSION

Differential expression and dysfunction of the exon junction complex (EJC) core protein has been reported in various cancers[7, 20–22]. RBM8A, a core component of this complex, is involved in multiple steps of transcription [8, 23]. In previous work, we found that *RBM8A* was overexpressed in 105 HCC tissue samples from patients in Guangxi, and that high *RBM8A* expression predicted poor prognosis [19]. In that work, we also showed *in vitro* that *RBM8A* promotes tumor cell migration and invasion in HCC by activating the EMT signaling pathway. To gain more detailed insights into the potential functions of *RBM8A* in HCC and its regulatory network, we performed bioinformatics analysis of public sequencing data to guide future research in HCC.

Early detection of HCC is a difficult problem that perplexes clinicians. Alpha-fetoprotein (AFP) has long been used as an indicator for early screening of HCC, but only approximately 70% of HCC patients are AFP-positive [24]. Therefore, new HCC markers are needed to improve early diagnosis. Analysis of transcriptional sequencing data from more than 1000 clinical samples in the GEO and TCGA databases comprising six geographic regions and ethnic HCC studies [25–29] confirmed that *RBM8A* mRNA levels and CNVs are significantly higher in HCC than in normal liver tissue. The fold change is similar across the various HCC studies and, while not large, ranks *RBM8A* among the top 3–4% of all genes upregulated in HCC, based on CNVs. We suggest that *RBM8A* overexpression occurs in many cases of HCC and deserves further clinical validation as a potential diagnostic and prognostic marker.

CNVs can have major genomic implications, disrupting genes and altering genetic content, leading to phenotypic differences [30]. Our study found that the copy number of *RBM8A* was increased in HCC, and that the major type of *RBM8A* alteration was amplification, which was associated with shorter survival. We speculate that altered *RBM8A* expression and *RBM8A* dysfunction in HCC may result from alterations in chromosomal structure. Since *RBM8A* plays several important physiological functions, its alteration may cause changes in various downstream signaling pathways. Indeed, neighboring gene networks close to *RBM8A* generally show different degrees of amplification in HCC. Related functional networks are involved in ribosome signaling, RNA transport, mRNA surveillance and spliceosome signaling. Thus, the network of *RBM8A* alterations is involved in the core node of post-transcriptional regulation, which is closely related to RNA splicing and protein translation. This is consistent with the physiological function of *RBM8A* [23, 31].

Enrichment analysis of target gene sets using GSEA can help reveal important networks of target kinases, miRNAs and transcription factors. Our results suggest that the functional network of *RBM8A* participates primarily in the spliceosome, ribosome, DNA replication and cell cycle. Like the mutation network, the functional network of *RBM8A* transcription is involved in genomic stability, gene expression and the cell cycle. These findings are consistent with the fact that *RBM8A* is critical for efficient and faithful splicing of a specific subset of short introns in mitotic cell cycle-related genes [32]. It is critical to understand how alteration in a protein important for ensuring normal transcription can lead to major dysfunction and even to cancer such as HCC.

Genomic instability and mutagenesis are fundamental characteristics of cancer cells, and kinases and their associated signaling pathways help stabilize and repair genomic DNA [33, 34]. We found that *RBM8A* in HCC is associated with a network of kinases including ATR, AURKB, and CDK1. These kinases regulate genomic stability, mitosis and the cell cycle [35, 36]. In fact, ATR is one of the core kinase regulators of genomic stability; it initiates cellular responses to genomic instability and repair, directly phosphorylating more than 1000 important substrates, including tumor suppressor gene p53 protein and cell cycle regulatory proteins [37]. ATR kinase inhibitors can kill tumor cells and synergize with the cell-killing effects of chemoradiotherapy [38]. In HCC, *RBM8A* may regulate DNA replication, repair, and cell cycle progression via ATR kinase.

In 2011, Hanahan et al. described the 10 hallmark features of tumors, with “continuous proliferation” at the top [39]. The most important reason is that abnormal expression of cell cycle regulatory factors in tumor cells leads to cell cycle disorder, which results in aberrant proliferation, decreased differentiation, decreased apoptosis, and rapid multiplication and development. E2F1 is one of the key links in the cell cycle regulation network [40]. Abnormal E2F1 expression is actively involved in the occurrence and development of HCC. Studies have shown that increased expression of E2F1 is associated with poor prognosis in HCC patients [41], and studies with a transgenic mouse model of E2F1-induced HCC showed that the network of transcription factors targeted by RBM8A is related to E2F1 [42]. Moreover, we showed previously that RBM8A activates the EMT signaling pathway, which E2F1 alters in a variety of solid tumors, including HCC [43, 44]. Therefore, our analyses suggest that E2F1 is an important target of RBM8A, and that RBM8A acts through this factor to regulate the cell cycle and proliferation capacity of HCC. Further studies should test this hypothesis.

Our study identified several miRNAs that were associated with *RBM8A*. These short (20–24 nt) non-coding RNAs, normally involved in post-transcriptional regulation of gene expression, can contribute to human carcinogenesis [45]. The particular miRNAs in our study have been linked to tumor proliferation, apoptosis, cell cycle, invasion, metastasis, drug resistance and angiogenesis [46–49]. In fact, miR-363, miR-25 and miR-299 can be used as diagnostic and prognostic markers of HCC [50–52]. While miR-363 regulates E2F Transcription Factor 3 to inhibit HCC migration and invasion [53], miR-367 and miR-32 participate in EMT progression [54, 55]. Dysregulation of these miRNAs is consistent with the phenotype of *RBM8A* overexpression in HCC from our previous work [19].

This study provides multi-level evidence for the importance of *RBM8A* in hepatocarcinogenesis and its potential as a marker in HCC. Our results suggest that *RBM8A* overexpression in HCC has far-reaching effects in genomic stability and at multiple steps of gene expression (DNA replication, RNA splicing and protein translation) and of the cell cycle. *RBM8A* is specifically related to several tumor-associated kinases (such as ATR), miRNAs (such as miRNA-363), and transcription factors (such as E2F1). This study uses online tools based on the most popular bioinformatics theories to perform target gene analyses on tumor data from public databases. Compared with traditional chip screening, this method has the advantages of large sample size, low cost, and simplicity. This enables large-scale HCC genomics research and subsequent functional studies.

At the same time, the TCGA database has limitations. One is that the TCGA LIHC samples contain three ethnic groups. The genetic background and etiology of HCC can differ significantly across ethnic groups. Another limitation is that the LIHC samples contain relatively few patients in stage 4, yet the clinical reality is that most HCC patients are first diagnosed when their disease is advanced and prognosis is extremely poor. Therefore, our results should be verified in clinical samples showing sufficient coverage of different ethnic groups and HCC stages. The third limitation is that transcriptome sequencing can detect only static mutations; it cannot directly provide information on protein activity or expression level. These questions should be addressed in follow-up studies using molecular biology techniques.

## MATERIALS AND METHODS

### Oncomine analysis

The mRNA expression and DNA copy number of *RBM8A* in HCC were analyzed within the Oncomine 4.5 database. Oncomine (www.oncomine.org), currently the world’s largest oncogene chip database and integrated data mining platform, contains 715 gene expression data sets and data from 86,733 cancer tissues and normal tissues [56]. This analysis drew on a series of HCC studies, including Roessler Liver 2, Chen Liver, Wurmbach Liver, Mas Liver, TCGA Liver and Guichard Liver studies [25–29]. *RBM8A* expression was assessed in HCC tissue relative to its expression in normal tissue, and differences associated with *p* < 0.01 were considered significant.

### UALCAN analysis

UALCAN uses TCGA level 3 RNA-seq and clinical data from 31 cancer types, which is an interactive web-portal to perform in-depth analyses of TCGA gene expression data [57]. One of the portal’s user-friendly features is that it allows analysis of relative expression of a query gene(s) across tumor and normal samples, as well as in various tumor sub-groups based on individual cancer stages, tumor grade or other clinicopathological features. UALCAN is publicly available at http://ualcan.path.uab.edu.

### c-BioPortal analysis

The cBio Cancer Genomics Portal (http://cbioportal.org) is an open-access resource for interactive exploration of multidimensional cancer genomics data sets, currently containing 225 cancer studies [58]. We used c-BioPortal to analyze *RBM8A* alterations in the TCGA LIHC sample. The search parameters included mutation, CNVs, and mRNA expression. The tab *OncoPrint* displays an overview of genetic alterations per sample in *RBM8A*. The tab *Network* visualizes the biological interaction network of *RBM8A* derived from public pathway databases, with color-coding and filter options based on the frequency of genomic alterations in each gene. Neighboring genes with alteration frequencies greater than 20% were included. We then performed GO and KEGG pathway enrichment analyses of the most frequently altered neighbor genes using the “clusterProfiler” package in R [59]. The GO annotation had three parts: cellular component (CC), biological process (BP), and molecular function (MF).

### LinkedOmics analysis

The LinkedOmics database (http://www.linkedomics.org/login.php) is a Web-based platform for analyzing 32 TCGA cancer-associated multi-dimensional datasets [60]. The *LinkFinder* module of LinkedOmics was used to study genes differentially expressed in correlation with *RBM8A* in the TCGA LIHC cohort (n=371). Results were analyzed statistically using Pearson’s correlation coefficient. The *LinkFinder* also created statistical plots for individual genes. All results were graphically presented in volcano plots, heat maps or scatter plots. The *LinkInterpreter* module of LinkedOmics performs pathway and network analyses of differentially expressed genes. The comprehensive functional category database in the Web-based Gene SeT AnaLysis Toolkit (WebGestalt) [61] was applied. Data from the *LinkFinder* results were signed and ranked, and GSEA was used to perform analyses of GO (CC, BP and MF), KEGG pathways, kinase-target enrichment, miRNA-target enrichment and transcription factor-target enrichment. The latter two network analyses were based on the Molecular Signatures Database (MSigDB) [62]. The rank criterion was an FDR < 0.05, and 500 simulations were performed.

### GeneMANIA analysis

GeneMANIA (http://www.genemania.org) is a flexible, user-friendly web interface for constructing protein-protein interaction (PPI) network, generating hypotheses about gene function, analyzing gene lists and prioritizing genes for functional assays [63]. The website can set the source of the edge of the network, and it features several bioinformatics methods: physical interaction, gene co-expression, gene co-location, gene enrichment analysis and website prediction. We used GeneMANIA to visualize the gene networks and predict function of genes that GSEA identified as being enriched in HCC: kinase ATR, mi-RNA 527 and transcription factor E2F1.

## SUPPLEMENTARY MATERIALS

Supplementary File
