# Supplementary material for: Expression and gene regulation network of RBM8A in hepatocellular carcinoma based on data mining
Source: Aging (Albany NY). 2019 Jan 22;11(2):423–47. doi: 10.18632/aging.101749 (PMC6366983; doi:10.18632/aging.101749)
Supplement: Supplementary File [file aging-11-101749-s001.pdf]

SUPPLEMENTARY MATERIALS

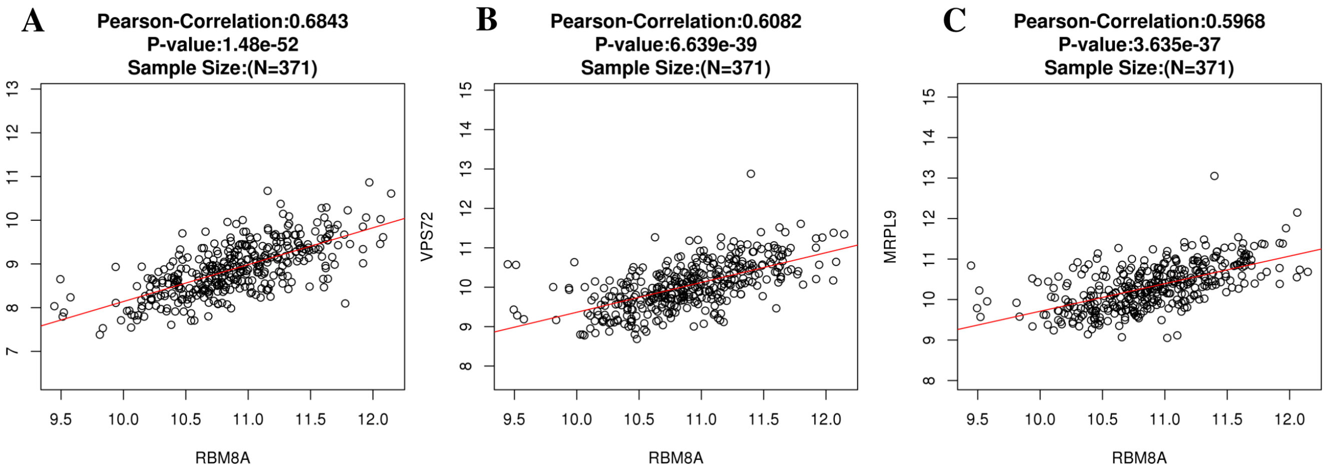

Supplementary Figure 1. Gene expression correlation analysis for *RBM8A, POLR3C, VPS72,* and *MRPL9* (LinkedOmics). The scatter plot shows Pearson correlation of *RBM8A* expression with expression of *POLR3C* (A), *VPS72* (B), and *MRPL9* (C).

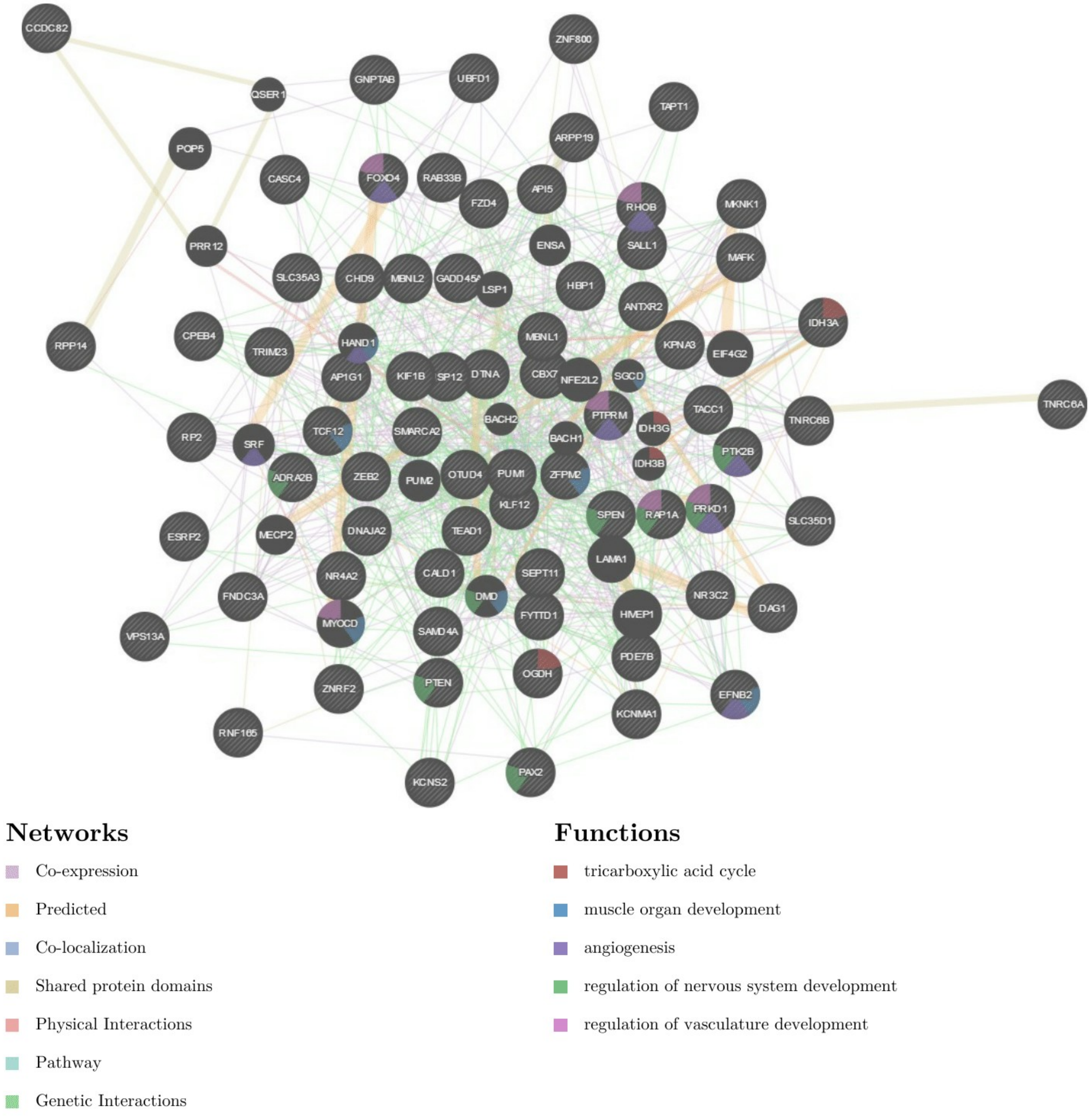

Supplementary Figure 2. Protein-protein interaction network of miRNA 527-target networks (GeneMANIA). Protein-protein interaction (PPI) network and functional analysis indicating the gene set that was enriched in the target networks of miRNA 527. Different colors of the network edge indicate the bioinformatics methods applied: co-expression, website prediction, co-localization, shared protein domains, physical interaction, pathway and genetic interactions. The different colors for the network nodes indicate the biological functions of the set of enrichment genes.

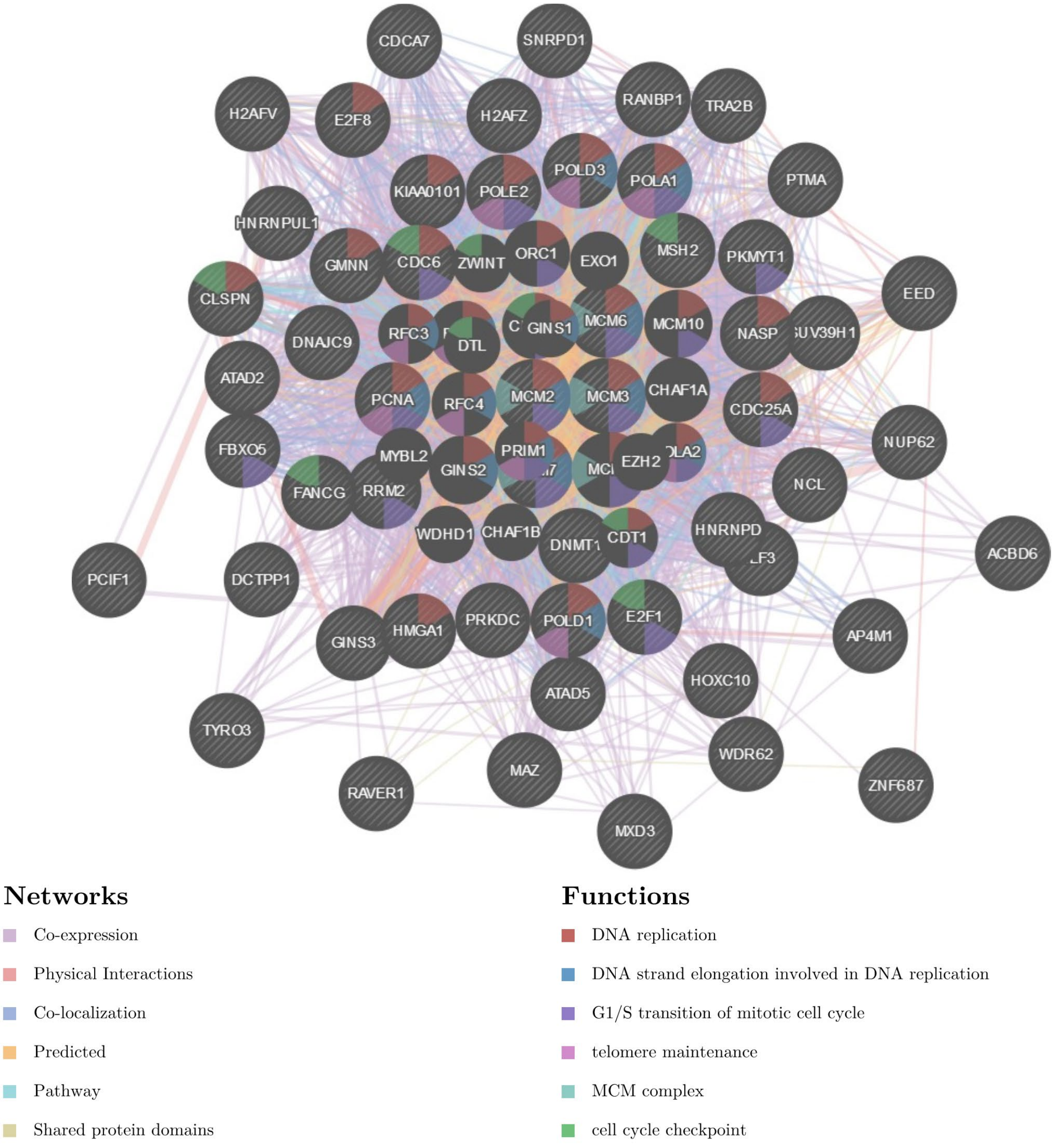

Supplementary Figure 3. Protein-protein interaction network of transcription factor E2F1DP1RB-target networks (GeneMANIA). Protein-protein interaction (PPI) network and functional analysis indicating the gene set that was enriched in the target networks of transcription factor E2F1DP1RB. Different colors of the network edge indicate the bioinformatics methods applied: co-expression, physical interaction, co-localization, website prediction, pathway and shared protein domains. The different colors for the network nodes indicate the biological functions of the set of enrichment genes.

Supplementary Table 1. Significantly enriched GO annotations (cellular components) of *RBM8A* in hepatocellular carcinoma (LinkedOmics).

Description	Leading EdgeNum	FDR	leadingEdgeGene
condensed chromosome	86	0	SMC4;AKAP8;NDC80;CDX2;CENPA;CENPB;SMC2;SPAG5;CENPE;CENPF;KIF2C;RCC1;TOPBP1;CHEK1;ZWINT;DMC1;DCTN3;CDCA5;CBX3;HUS1B;SPC24;DDX11;CENPV;FANCD2;SKA1;SKA3;NCAPH;ITGB3BP;PES1;NSL1;SMC1B;H2AFX;HMGB1;HMGB2;BIRC5;INCENP;CENPW;MAD2L1;MKI67;NEK2;DYNC1LI1;PLK1;ERCC6L;PPP1CC;ZWILCH;HJURP;NUP133;SPC25;RAD1;RAD9A;RAD51;RAD51C;RANGAP1;BLM;CENPK;NCAPG;CENPH;BRCA1;AURKA;SUV39H1;BUB1;BUB1B;TOP2A;TUBG1;UBE2I;ZNF207;CENPM;NUP37;CENPO;NUP85;DSN1;SEH1L;NUF2;MAD1L1;DCTN5;BANF1;CCNB1;TOP3B;RSPH1;BOD1;BUB3;AURKB;H2AFY;KNTC1;CKAP5;NCAPD2
replication fork	30	0	POLD3;CHEK1;DNMT1;POLA2;UHRF1;H2AFX;MCM3;SMARCAL1;PCNA;GINS2;POLE3;POLA1;POLD1;POLE2;TIPIN;MCM10;PRIM1;PRIM2;RAD51C;RFC2;RFC4;RFC5;RPA1;RPA3;XPA;XRCC2;XRCC3;PIF1;CDC45;GINS4
spliceosomal complex	68	0	SF3B4;CWC27;ADAR;PPIH;USP39;TXNL4A;SF3A3;SF3B2;LSM6;U2AF2;HNRNPA1L2;DQX1;U2AF1L4;HNRNPA3;RALY;SNW1;TFIP11;PRPF6;PRPF31;LSM3;RBMX;HNRNPA1;HNRNPA2B1;HNRNPC;HNRNPH1;HNRNPH3;HNRNPU;MAGOH;HNRNPM;CRNKL1;RBMX2;PPIL1;LSM7;GPATCH1;MAGOHB;PRCC;RBM22;XAB2;LSM2;SNRNP70;SNRPA;SNRPA1;SNRPB;SNRPB2;SNRPC;SNRPD1;SNRPD2;SNRPD3;SNRPE;SNRPF;SNRPG;SF1;RNF113A;SF3A2;DGCR14;SF3B5;WDR83;DHX16;PRPF38A;RBM17;SART1;PRPF4;PRPF3;EFTUD2;SNRNP40;DDX23;EIF4A3;RBM8A
spindle	112	0	KIF20A;TACC3;SPAG5;CENPE;CENPF;TUBGCP2;JTB;CBX1;TOPBP1;KATNA1;BIRC8;DCTN3;CBX3;HAUS1;CLTA;CSNK1D;KIF18B;CKAP2L;DCTN1;DDX11;ECT2;CENPV;ERCC2;SKA1;SKA3;MAPRE1;TPX2;HAUS5;NUP62;KIF4A;HEPACAM2;POC1A;ASPM;FBXO5;CKAP2;PRPF19;WDR62;RACGAP1;GPSM2;HSPB1;BIRC5;TUBB8;INCENP;KIF2A;KIF11;KIFC1;KIF22;ARL3;MAD2L1;NEK2;NPM1;DYNC1LI1;NUSAP1;PLK1;PPP2R3C;CDCA8;PLEKHG6;MAP1S;HAUS7;YEATS2;CDK5RAP2;RCC2;KIF15;RANGAP1;AGBL5;RPS3;MMS19;AURKA;BUB1B;TPR;TTK;TUBG1;VRK1;ZNF207;ALMS1;HAUS3;SHCBP1;NUP85;DSN1;FAM83D;KIF18A;CAPG;FAM110A;MAD1L1;GSG2;RAB11FIP4;PSRC1;KLHL22;WDR73;DYNLL1;HDAC3;CDC16;CCNB1;RSPH1;PRC1;UNC119;ARHGEF2;AURKB;HAUS8;KIF23;KIF20B;MAD2L1BP;ESPL1;KNTC1;DLGAP5;CKAP5;CDK1;CEP350;CDC6;CDC20;KIF14;CDC25B

Abbreviations: LeadingEdgeNum, the number of leading edge genes; FDR, false discovery rate from Benjamini and Hochberg from gene set enrichment analysis (GSEA).

Supplementary Table 2. Significantly enriched GO annotations (biological processes) of *RBM8A* in hepatocellular carcinoma (LinkedOmics).

Description	Leading EdgeNum	FDR	LeadingEdgeGene
cell cycle checkpoint	89	0	CDK2;NDRG1;NDC80;MAD2L2;CENPF;TOPBP1;CHEK1;ZWINT;CHEK2;E2F7;CSNK2A1;EME1;E2F1;E2F4;FANCG;CNOT10;HINFP;FBXO4;PRPF19;SFN;H2AFX;HRAS;PRMT1;MAD2L1;MSH2;CNOT3;NPM1;PCNA;DYNC1LI1;WAC;LCMT1;TRIAP1;GTSE1;DTL;UIMC1;PLK1;TIPIN;ZWILCH;RFWD3;FANCI;PRCC;CDK5RAP2;PCID2;CENPJ;PRKDC;BAX;RAD1;RBBP8;RPA2;RPS6;RPS27A;CLSPN;BLM;DCLRE1B;BRCA1;AURKA;BUB1;BUB1B;TFDP2;TOP2A;TTK;UBA52;XRCC3;ZNF207;RNASEH2B;WDR76;CEP63;CDK5RAP3;HMGA2;CDT1;CDC45;GSG2;BRIP1;ATRIP;THOC5;CCNB1;CCNE2;ZNF830;ZW10;BUB3;AURKB;BRE;MAD2L1BP;MDC1;KNTC1;CDK1;CDC5L;CDC6;CDC25C
DNA replication	100	0	CHAF1A;RNASEH2A;POLD3;POLQ;EHMT2;DBF4;CHEK1;SUPT16H;CHEK2;POLG2;PARP1;E2F7;EME1;ESCO2;DDX11;FEN1;KIN;GTPBP4;POLA2;CACYBP;POLL;STOML2;HMGA1;HRAS;STRA8;LIG1;MCM2;MCM3;MCM4;MCM5;MCM6;MCM7;NAP1L1;NASP;SMARCAL1;GMNN;PCNA;DTL;CINP;GINS2;RTEL1;PLA2G1B;POLE3;POLA1;POLD1;POLD2;POLE2;TEX10;TIPIN;PPP2R1A;MCM10;PRIM1;PRIM2;RAD1;RAD9A;RAD51;RBBP7;RFC2;RFC4;RFC5;RPA1;RPA3;RRM1;RRM2;CHTF18;CLSPN;TSPYL2;BLM;SET;SHC1;NUCKS1;GINS3;BRCA1;SSRP1;TOP2A;TTF1;SLBP;DSCC1;E2F8;CDT1;CHAF1B;CDC7;CDC45;ATRIP;RPAIN;ING5;GINS4;MCM8;CCNA2;TIMELESS;CCNE1;EXO1;RECQL4;KIAA0101;CDK1;GINS1;CDC6;CDC25A;CDC25C;THOC1
mRNA processing	149	0	PQBP1;CDK7;SF3B4;CWC27;ADAR;PRMT5;PPIH;PRPF8;KHDRBS1;USP39;CPSF4;TXNL4A;RNPS1;SF3A3;HNRNPA0;SF3B2;CPSF6;HNRNPUL1;LSM6;U2AF2;TSEN15;RAVER1;LSM11;HNRNPA1L2;CSTF1;CSTF2;DQX1;DHX9;U2AF1L4;ERCC2;HNRNPA3;CASC3;PUF60;RALY;NCBP2;SNW1;KIN;PDCD11;KDM1A;JMJD6;TARDBP;TFIP11;PRPF6;FUS;ZNF473;PRPF31;DAZAP1;AKAP8L;GPKOW;LSM3;RBMX;PRPF19;TSEN54;GTF2F1;GTF2H4;CPSF1;HMX2;HNRNPA1;HNRNPA2B1;HNRNPC;HNRNPD;HNRNPH1;HNRNPH3;HNRNPL;HNRNPU;MAGOH;HNRNPM;H2AFB1;NONO;GEMIN4;DDX47;CRNKL1;SRRT;RBMX2;PPIL1;LSM7;CPSF3;POLR2D;POLR2F;POLR2G;POLR2H;POLR2I;PAF1;GPATCH1;MAGOHB;RAVER2;PSPC1;WDR33;RBM38;RBM22;RNF20;XAB2;PTBP1;LSM2;SCAF1;UBL5;RBM4;SAFB;SFPQ;CPSF4L;TRA2B;SNRNP70;SNRPA;SNRPA1;SNRPB;SNRPB2;SNRPC;SNRPD1;SNRPD2;SNRPD3;SNRPE;SNRPF;SNRPG;SUPT5H;SUPT6H;SF1;RNF113A;SLBP;TSEN34;RBM42;GEMIN7;FIP1L1;SF3A2;DGCR14;RBM10;SF3B5;FAM103A1;WDR83;THOC3;SARNP;DHX16;BUD13;PHF5A;PRPF38A;RBM17;THOC5;KHSRP;CCNB1;SART1;PRPF4;PRPF3;EFTUD2;SNRNP40;DDX23;RBM39;SAFB2;EIF4A3;RBM8A;THOC1
fatty acid metabolic process	133	0	LYPLA1;ACAA2;CES1;PPARGC1A;ACOT2;SLC27A5;SLC27A4;SLC27A2;ERLIN2;CYP2U1;APOA5;ACSM2A;ACOT12;CPT2;CRAT;PTGR2;CYP1A1;CYP1A2;CYP1B1;CYP2A6;CYP2A7;CYP2A13;CYP2C19;CYP2C8;CYP2C9;CYP2J2;CYP4A11;AKR1C2;DECR1;MMAA;DLAT;DLD;ECHS1;EDN1;EHHADH;EPHX2;AKT2;ETFA;ETFDH;FAAH;ACSL1;ALDH3A2;PTGR1;WDTC1;LPIN1;ACSL6;SIRT1;MLYCD;AMACR;MTOR;HACL1;HIBCH;GHR;ACAD8;ANGPTL3;ZADH2;CYP4V2;ACAA1;HADHB;HADH;ACACB;HSD17B4;ACADL;ACADM;ACSM2B;ACADS;

Description	Leading EdgeNum	FDR	LeadingEdgeGene
			ACADSB;INSIG1;IRS1;ACADVL;IVD;ACAT1;LIPE;LPL;CYP4F3;MGST2;MUT;MYO5A;PNPLA8;PCCA;ACOX1;CRYL1;ABHD5;INSIG2;HSD17B12;PRKAG2;PDHA1;PDK1;PDK2;PDK4;ACSL5;PEX13;PLA2G5;HAO1;POR;PPARA;AUH;ACSM5;OXSM;PDPR;AVPR1A;THNSL2;ECHDC2;OLAH;PECR;BDH2;BAAT;PDP2;GPAM;ABCD3;ELOVL5;ACSM3;SCP2;ACOT1;ACOT6;STAT5B;TNFRSF1A;ELOVL6;ALDH5A1;ADIPOR2;PDHX;ACAD10;ACOX2;SESN2;LONP2;ACAD11;MCEE;CBR4;CYP4F2;IRS2;CBR1;SGPL1;LPIN2

Abbreviations: LeadingEdgeNum, the number of leading edge genes; FDR, false discovery rate from Benjamini and Hochberg from gene set enrichment analysis (GSEA).

Supplementary Table 3. Significantly enriched GO annotations (molecular functions) of *RBM8A* in hepatocellular carcinoma (LinkedOmics).

Description	Leading EdgeNum	FDR	LeadingEdgeGene
structural constituent of ribosome	85	0	RPL35;RPL39L;MRPL52;MRPL55;RPL13A;RPL36;RPSA;RPL10A;MRPL4;MRPL51;MRPS17;MRPS23;MRPS21;MRPS18A;MRPL47;RPL5;RPL6;RPL7;RPL7A;RPL8;RPL10;RPL17;RPL18;RPL18A;RPL19;RPL21;RPL22;RPL23A;MRPL23;RPL24;RPL26;RPL27;RPL30;RPL27A;RPL28;RPL31;RPL32;RPL35A;RPL37;RPL37A;RPL38;RPL39;RPL41;RPL36A;RPLP0;RPLP1;RPLP2;MRPS12;RPS2;RPS3;RPS3A;RPS4X;RPS5;RPS6;RPS7;RPS8;RPS9;RPS10;RPS11;RPS12;RPS13;RPS14;RPS15;RPS15A;RPS16;RPS17;RPS18;RPS19;RPS21;RPS23;RPS24;RPS27;RPS27A;RPS29;MRPS14;MRPL14;MRPS24;MRPS5;MRPL11;MRPL9;UBA52;DAP3;MRPL24;RPL23;MRPL33
monooxygenase activity	37	0	COQ7;CYP2U1;CYP1A1;CYP1A2;CYP1B1;CYP2A6;CYP2A7;CYP2C19;CYP2C8;CYP2J2;CYP3A4;CYP4A11;CYP8B1;CYP11A1;CYP17A1;CYP26A1;DBH;AKR1C2;NLRP11;FMO2;FMO3;FMO4;CYP4X1;CYP4A22;CYP4V2;CYP4F3;NOS1;NOS3;PAH;COQ6;CYP39A1;MICAL3;CYP4F11;CYP3A43;CYP4F2;CYP7B1;MICAL2
oxidoreductase activity, acting on CH-OH group of donors	63	0	AKR1A1;ME3;HIBADH;ADH1A;ADH1B;ADH1C;ADH4;ADH5;ADH6;ADHFE1;PTGR2;CYB5A;AKR1C1;AKR1C2;EHHADH;LDHD;ALDH3A1;ALDH3A2;PTGR1;AKR7A3;KDSR;GPD1;ZADH2;HADHB;HADH;AOX1;HSD11B1;HSD17B3;HSD17B4;IDH1;IDH2;IDH3A;CYP4F3;MDH1;ME1;CRYL1;RDH11;HSD17B12;HSD17B11;DCXR;HAO1;CHDH;BDH2;RDH14;RDH5;BDH1;SORD;SRD5A2;D2HGDH;XDH;KCNAB1;VKORC1;L2HGDH;GLYR1;CBR4;KCNAB2;CYP4F2;RDH16;HSD17B6;CBR1;DHRS3;GRHPR;H6PD
cofactor binding	105	0	AHCYL1;ME3;SDS;HIBADH;PROSC;ADH4;CRY2;CRYM;CRYZ;CTH;DECR1;DHODH;CYB5R3;DLD;ABAT;LDHD;ETFA;ALAS1;ETFDH;ALDH1A3;FMO2;FMO3;FMO4;AHCYL2;SIRT5;SIRT1;NNT;MMACHC;HACL1;ACAD8;GCLC;GOT2;GPD1;GPT;HADH;HDC;AOC2;HMGCL;ACADL;GADL1;ACADM;IDH1;IDH3A;ACADS;ACADSB;ACADVL;IVD;ACAT1;IYD;LMO2;MAOB;ME1;ALDH6A1;NOS1;NOS3;OGDH;ACOX1;COQ6;CRYL1;AADAT;CSAD;WWOX;HAO1;POR;PNPO;THNSL2;ASPDH;DHTKD1;OGDHL;BDH2;MICAL3;QDPR;SCP2;SRR;SDHA;SDHB;SDHD;SHMT1;AGXT2;SORD;SRD5A1;SUOX;TAT;D2HGDH;XDH;KCNAB1;VKORC1;ACBD4;THNSL1;COQ10B;ACAD10;ACOX2;ACAD11;GLYR1;ACCS;CAT;GPT2;CBR4;AOC3;SGPL1;ACBD5;OXNAD1;GRHPR;H6PD;MICAL2

Abbreviations: LeadingEdgeNum, the number of leading edge genes; FDR, false discovery rate from Benjamini and Hochberg from gene set enrichment analysis (GSEA).

Supplementary Table 4. Significantly enriched KEGG pathway annotations of *RBM8A* in hepatocellular carcinoma (LinkedOmics).

Description	Leading EdgeNum	FDR	LeadingEdgeGene
Spliceosome	62	0	PQBP1;SF3B4;PPIH;USP39;TXNL4A;SF3A3;SF3B2;LSM6;U2AF2;HNRNPA1L2;U2AF1L4;HNRNPA3;PUF60;NCBP2;SNW1;PRPF6;PRPF31;LSM3;RBMX;HNRNPA1;HNRNPC;HNRNPU;HSPA6;MAGOH;HNRNPM;CRNKL1;PPIL1;LSM7;MAGOHB;RBM22;XAB2;ISY1;LSM2;RP9;TRA2B;SNRNP70;SNRPA;SNRPA1;SNRPB;SNRPB2;SNRPC;SNRPD1;SNRPD2;SNRPD3;SNRPE;SNRPF;SNRPG;U2AF1;SF3A2;SF3B5;THOC3;DHX16;PRPF38A;RBM17;SART1;PRPF4;PRPF3;EFTUD2;SNRNP40;DDX23;EIF4A3;RBM8A
Ribosome	79	0	RPL35;RPL13A;RPL36;RPSA;RPL10A;MRPL4;MRPS17;MRPS21;MRPS18A;RPL5;RPL6;RPL7;RPL7A;RPL8;RPL10;RPL17;RPL18;RPL18A;RPL19;RPL21;RPL22;RPL23A;MRPL23;RPL24;RPL26;RPL27;RPL30;RPL27A;RPL28;RPL31;RPL32;RPL35A;RPL37;RPL37A;RPL38;RPL39;RPL41;RPL36A;RPLP0;RPLP1;RPLP2;MRPS12;RPS2;RPS3;RPS3A;RPS4X;RPS5;RPS6;RPS7;RPS8;RPS9;RPS10;RPS11;RPS12;RPS13;RPS14;RPS15;RPS15A;RPS16;RPS17;RPS18;RPS19;RPS21;RPS23;RPS24;RPS25;RPS26;RPS27;RPS27A;RPS29;MRPS14;MRPL14;MRPS5;MRPL11;MRPL9;UBA52;MRPL24;RPL23;MRPL33
DNA replication	24	0	RNASEH2A;POLD3;FEN1;POLA2;LIG1;MCM2;MCM3;MCM4;MCM5;MCM6;MCM7;PCNA;POLE3;POLA1;POLD1;POLE2;PRIM1;PRIM2;RFC2;RFC4;RFC5;RPA1;RPA3;RNASEH2B
Cell cycle	51	0	CDK4;CDK7;CDKN2C;PTTG2;DBF4;YWHAQ;CHEK1;CHEK2;E2F1;E2F2;E2F4;CDC26;SMC1B;ANAPC4;HDAC1;HDAC2;MAD2L1;MCM2;MCM3;MCM4;MCM5;MCM6;MCM7;PCNA;ANAPC5;ANAPC7;ANAPC11;PLK1;PRKDC;BUB1;BUB1B;TFDP2;TTK;CDC7;CDC45;CDC16;CCNA2;CCNB1;CCNE1;PKMYT1;CCNB2;CCNE2;BUB3;PTTG1;ESPL1;CDK1;CDC6;CDC20;CDC25A;CDC25B;CDC25C
RNA transport	50	0	POM121C;POP7;PRMT5;TACC3;RPP30;RPP38;POP4;RNPS1;EIF2B1;EIF2S3;EIF4A1;CASC3;NCBP2;NUP205;NUP210;NUP188;NUP62;NXT1;MAGOH;GEMIN4;MAGOHB;ELAC1;NUP133;XPO5;RAN;RANGAP1;UPF3A;SUMO2;TPR;UBE2I;NUP37;GEMIN7;RPP21;NUP85;NUP214;THOC3;RAE1;THOC5;EIF3B;EIF3D;EIF3F;EIF3G;EIF2B5;EIF2S2;EIF4E2;NUP155;NUP93;EIF4A3;RBM8A;THOC1
mRNA surveillance pathway	29	0	CPSF4;RNPS1;CPSF6;MSI2;CSTF1;CSTF2;CSTF3;CASC3;NCBP2;SMG5;DAZAP1;NXT1;CPSF1;MAGOH;MSI1;CPSF3;PPP1CA;PPP1CC;PPP2R3C;MAGOHB;PPP2R1A;PPP2R5D;WDR33;UPF3A;FIP1L1;SYMPK;EIF4A3;SMG7;RBM8A

Abbreviations: LeadingEdgeNum, the number of leading edge genes; FDR, false discovery rate from Benjamini and Hochberg from gene set enrichment analysis (GSEA).

Supplementary Table 5. Significantly enriched kinase-target networks of *RBM8A* in hepatocellular carcinoma (LinkedOmics).

Geneset	LeadingEdgeGene
Kinase_ATR	DBF4;CHEK1;CHEK2;DAXX;E2F1;EWSR1;FANCD2;FARSA;H2AFX;MCM2;MCM3;CNOT2;SMARCAL1;GINS2;FANCI;TDP1;RAD1;RAD9A;CHTF18;CLSPN;BLM;DCLRE1C;MARCKSL1;BRCA1;XPA;XRCC3;ATRIP;MDC1
Kinase_AURKB	NDC80;RBM14;MYBBP1A;CENPA;KIF2C;SUPT16H;CDCA5;HIST2H3C;CDCA2;KIF4A;ATXN10;CKAP2;RBMX;RACGAP1;H3F3A;HMGN2;BIRC5;HSP90AB1;MKI67;NUSAP1;PLK1;CDCA8;PRKDC;PARD3;RBM3;RPL8;RPL21;RPS10;DEK;SHCBP1;DSN1;HIST1H3C;AURKB;KIF23
Kinase_CDK1	BCL2L11;CDK7;SCML2;CENPA;SPAG5;PTTG2;SEPT9;CD3EAP;KIF2C;CHEK1;CDCA5;U2AF2;CSNK2A1;CSNK2B;DCTN1;DNMT1;E2F1;ECT2;EEF1D;EZH2;FANCG;FEN1;TPX2;FOXM1;CARHSP1;CKAP2;FBXO43;UHRF1;TFPT;PYCR2;ANAPC4;HIST1H1E;HCFC1;HMGA1;BIRC5;FOXK2;KIF11;KIF22;STMN1;LBR;LIG1;LMNA;LMNB1;MCM7;MDM4;MKI67;NCL;NUSAP1;DTL;ANAPC11;PI4KB;ERCC6L;PPP1CA;CEP55;PBK;NSFL1C;PTPN2;RAD9A;RANGAP1;RFC2;RFC4;RFC5;RPS3;RRM2;BLM;NCAPG;NUCKS1;BRCA1;SSR1;SUPT5H;TCOF1;BUB1;BUB1B;TK1;TMPO;TOP2A;TPR;UBE2I;USP1;SLBP;CDC7;GSG2;LMNB2;GMPS;CDC16;CCNB1;PRC1;KIF20B;ESPL1;DLGAP5;UBAP2L;CDC20;CDC25A;CDC25B;CDC25C
Kinase_CDK2	CDK7;SCML2;CDX2;CENPF;CHD4;HNRNPUL1;CHEK1;EGLN2;C17orf49;CSNK2A1;CSNK2B;DNMT1;E2F1;E2F2;CRTC2;EZH2;RALY;SNW1;TPX2;FOXM1;ANKLE2;NCAPH;SNAPIN;CDC26;XRCC6;MTHFD1L;PRPF31;ERAL1;PELP1;POLL;DBNL;UHRF1;GTF2F1;PYCR2;ANAPC4;HIST1H1E;HCFC1;HMGA1;HNRNPD;HNRNPH1;FOXK2;ILF3;IMPDH2;KIF22;STMN1;LIG1;LIG3;MARCKS;MCM2;MCM3;MCM4;MCM7;MKI67;MYBL2;NCL;NOSIP;CDK16;ANAPC5;ANAPC7;DTL;ANAPC11;POU2F1;PPP1CA;C9orf40;NUP133;DMAP1;PTHLH;AARS2;USP37;PTPN2;PYCR1;RAD9A;DPF2;RPS3;RPS27;RRM2;TSPYL2;BLM;NUCKS1;SNRNP70;BRCA1;SUPT5H;SUPT6H;TBCE;TCF3;TCOF1;TK1;TPR;TSN;XRCC1;ZYX;CDT1;DIAPH3;CDC7;ATRIP;ING5;LMNB2;ARHGAP19;GMPS;CDC16;CCNA2;CCNE1;PRPF3;MDC1;EIF4A3;DLGAP5;SETDB1;CDC6;CDC20;CDC25C
Kinase_CHEK1	TRIM28;RBM14;MYBBP1A;POP4;TLK2;CHEK1;RAVER1;CSNK2B;E2F6;EPRS;FANCD2;FANCE;FEN1;RALY;RRP12;XRCC6;RACGAP1;H3F3A;HNRNPA2B1;KIFC1;KPNA2;LAMC1;LIG1;LMNA;MAD2L1;MCM3;MCM5;MDM4;MKI67;HNRNPM;PPP2R5D;MAP2K2;RAD51;RPL19;CLSPN;BLM;RBM42;BUD13;TNK1;PRPF3;ARHGEF2;AURKB;MDC1;CDK1;CDC25A;CDC25B;CDC25C

Abbreviations: LeadingEdgeNum, the number of leading edge genes; FDR, false discovery rate from Benjamini and Hochberg from gene set enrichment analysis (GSEA).

Supplementary Table 6. Significantly enriched miRNA-target networks of *RBM8A* in hepatocellular carcinoma (LinkedOmics).

Geneset	LeadingEdgeGene
CTTTGCA, MIR-527	MBNL2;DNAJA2;ARPP19;RPP14;KLF12;CASC4;ANTXR2;ADRA2B;DAG1;AP1G1;GADD45A;ZNF800;DTNA;EFNB2;TAPT1;PTK2B;USP12;ZNRF2;FNDC3A;SPEN;SAMD4A;KIF1B;TNRC6B;SLC35D1;VPS13A;ZFPM2;SLC35A3;CBX7;HBP1;PDE7B;TNRC6A;IDH3A;TRIM23;KCNMA1;KCNS2;KPNA3;RHOB;MBNL1;FOXO4;NR3C2;NR4A2;RNF165;OGDH;PAX2;OTUD4;SEPT11;PRKD1;UBFD1;PTEN;PTPRM;RAP1A;RP2;SALL1;SMARCA2;TACC1;TCF12;TEAD1;GNPTAB;MAFK;CCDC82;CALD1;ESRP2;CHD9;CPEB4;FZD4;RAB33B;FYTTD1;API5;MKNK1;PUM1;ZEB2
CCCACAT, MIR-299-3P	APBB3;CUX1;AP1G1;CYB5R3;MAPRE3;FBXO33;IFIT1;ITGAV;KCNMA1;MUC4;PARP6;ZNFX1;ARRDC3;RAB6A;ABCE1;PDLIM2;TCF4;TIMP3;UBE2H;TRPM3;RAB6C;RUNX1T1;CD164
GTGCAAT, MIR-25, MIR-32, MIR-92, MIR-363, MIR-367	FRY;TOB1;ADAM10;NFAT5;IQGAP2;FCHO2;LIN54;CPEB2;ADM;SESN3;CNTN4;DMXL1;S1PR1;PIKFYVE;FHL2;CPEB3;GOLGA8A;PHLPP2;PDZD2;DOCK9;MORC3;NECAP1;LATS2;NPTN;GOLGA4;GRIA3;BAZ2B;HIVEP1;RBPMS2;INSIG1;ITGAV;MYO18A;SMAD6;MAN2A1;DNAJB9;MYO1B;NFIA;NFIX;POLK;C11orf24;SLC38A2;OTUD4;BSDC1;UBE2W;FBXW7;PRKCE;ADAMTSL3;FAM160B1;REV3L;GPBP1L1;BMPR2;GRAMD3;SSFA2;TCF21;DYNLT3;TEAD1;TGIF1;UGP2;LUZP1;PPCS;CPEB4;SGPP1;SNN;DNAJC30;GLYR1;PIK3R3;CBFA2T3;KAT2B;CCNC;HERC2;KALRN;ARRDC4;DLGAP2;FNIP1;KIAA0430;EDEM1;ZEB2;TECPR2
GACAATC, MIR-219	PTPRU;PPARGC1A;AKAP13;FMNL2;SLC31A1;CPEB2;SH3D19;FAM91A1;ZNF800;UBR1;ELK1;EPHA4;ERG;ESR1;ZCCHC24;RBM24;CPEB3;KIF1B;DDAH1;ELMOD2;CCDC28A;FBXO3;AFF4;INSIG1;SNRK;CC2D1A;SOX6;AGPAT3;ERGIC1;RNF6;ITSN1;FBXL17;TACC1;KLF9;TGFBR2;NR2C2;UBE3A;SLC30A4;NRIP1;FZD4;TRAF7;KBTBD8;CGNL1;CBFA2T3;CD164;SYNGAP1;HOMER2;LAPTM4A;TSC22D2
ACTTTAT, MIR-142-5P	APBB3;SORBS1;NFAT5;BVES;MGAT4B;FCHO2;CLCN3;C14orf28;COL4A3;CPEB2;FAM91A1;AP1G1;MIER3;AHR;UBR1;ELAVL2;EPAS1;HIPK1;ETV1;JMJD1C;CPEB3;FNDC3A;SPEN;DCUN1D4;TBC1D9;ZCCHC14;ZFPM2;RHOQ;FOS;RALGAPA1;C2CD2;GCH1;NPAS4;AFF4;MAT2B;NRG1;CHSY3;LUZP2;IGF1;MKLN1;NFE2L2;RNF165;CDK17;MBIP;BTBD1;FAM134B;OTUD4;DNAJC25;CDC37L1;SLC17A7;GOPC;PTPN4;REV3L;SCOC;SDCBP;SNX16;ABCG4;PLEKHA3;BNIP2;TCF12;TGFBR2;KLF10;TLE4;ZFP36;ZFYVE21;RNF128;SPSB1;CHD9;CPEB4;FAM107B;ZNF503;RERG;DCHS1;SLC4A4;NRP1;SYNJ1;CCNT2;ZBTB47;TAOK2;UBE4A;SLC25A27;ADAMTS1;UBE3C;HERPUD1;TSC22D2

Abbreviations: LeadingEdgeNum, the number of leading edge genes; FDR, false discovery rate from Benjamini and Hochberg from gene set enrichment analysis (GSEA).

Supplementary Table 7. Significantly enriched transcription factor-target networks of *RBM8A* in hepatocellular carcinoma (LinkedOmics).

Geneset	LeadingEdgeGene
V$E2F1DP1RB_01	POLD3;HNRNPUL1;CLTA;SEZ6;RAVER1;E2F7;ZNF565;DNMT1;E2F1;EIF4A1;THAP8;EZH2;DNAJC9;ZIM2;NUP62;POLA2;INTS7;IL4I1;FBXO5;WDR62;ATAD2;H2AFZ;HMGN2;HNRNPA1;HNRNPD;HOXC10;RBPJ;ILF3;IMPDH2;STMN1;LIG1;MAZ;MCM2;MCM3;MCM6;MCM7;MSH2;NASP;NCL;GMNN;APH1A;PCNA;TRMT6;POLA1;POLD1;POLE2;ANKHD1;PPP1CC;OTUD7B;ZNF687;USP37;RAD51;RRM2;CLSPN;GINS3;SNRPD1;SOAT1;TMPO;TYRO3;DCTPP1;E2F8;ATAD5;CDT1;MXD3;CDCA7;ACBD6;MCM8;FIZ1;EED;PKMYT1;AP4M1;KIAA0101;ARHGAP11A;CDK1;CDC6;CDC25A
V$E2F1DP1_01	POLD3;CD3EAP;HNRNPUL1;CBX3;RAVER1;E2F7;ZNF565;DNMT1;E2F1;EIF4A1;THAP8;EZH2;FANCD2;FANCG;DNAJC9;ZIM2;NUP62;IL4I1;FBXO5;WDR62;ATAD2;H2AFZ;HMGA1;HNRNPA2B1;HNRNPD;HOXC10;ILF3;STMN1;MAZ;MCM2;MCM3;MCM4;MCM6;MCM7;MSH2;NASP;NCL;PAX6;GMNN;APH1A;PCNA;TRMT6;POLA1;POLD1;POLE2;PRKDC;OTUD7B;PTMA;ZNF687;USP37;RANBP1;RRM2;PCIF1;CLSPN;TRA2B;GINS3;GPBP1;SNRPD1;SOAT1;SUV39H1;TMPO;TYRO3;DCTPP1;E2F8;ATAD5;MXD3;CDCA7;ACBD6;MCM8;PHF5A;FIZ1;EED;PKMYT1;AP4M1;H2AFV;KIAA0101;ARHGAP11A;CDK1;CDC6;CDC25A
V$E2F1DP2_01	POLD3;CD3EAP;HNRNPUL1;CBX3;RAVER1;E2F7;ZNF565;DNMT1;E2F1;EIF4A1;THAP8;EZH2;FANCD2;FANCG;DNAJC9;ZIM2;NUP62;IL4I1;FBXO5;WDR62;ATAD2;H2AFZ;HMGA1;HNRNPA2B1;HNRNPD;HOXC10;ILF3;STMN1;MAZ;MCM2;MCM3;MCM4;MCM6;MCM7;MSH2;NASP;NCL;PAX6;GMNN;APH1A;PCNA;TRMT6;POLA1;POLD1;POLE2;PRKDC;OTUD7B;PTMA;ZNF687;USP37;RANBP1;RRM2;PCIF1;CLSPN;TRA2B;GINS3;GPBP1;SNRPD1;SOAT1;SUV39H1;TMPO;TYRO3;DCTPP1;E2F8;ATAD5;MXD3;CDCA7;ACBD6;MCM8;PHF5A;FIZ1;EED;PKMYT1;AP4M1;H2AFV;KIAA0101;ARHGAP11A;CDK1;CDC6;CDC25A
V$E2F1_Q3	POLD3;PLK4;CD3EAP;HNRNPUL1;RAVER1;E2F7;SIX5;ZNF565;DNMT1;E2F1;THAP8;EZH2;DOLK;DNAJC9;ZIM2;NUP62;IL4I1;FBXO5;PELP1;WDR62;GPS2;ATAD2;H2AFZ;HMGN2;HNRNPD;HOXC10;ILF3;STMN1;MAZ;MCM2;MCM3;MCM6;MCM7;MSH2;NASP;NCL;CNOT3;PAX6;GMNN;APH1A;PCNA;TRMT6;POLA1;POLD1;POLE2;ANKHD1;TIPIN;OTUD7B;KIF15;ZNF687;USP37;RRM2;CLSPN;GINS3;SNRPD1;SOAT1;TMPO;TYRO3;USP1;DCTPP1;E2F8;ATAD5;MXD3;CDCA7;ACBD6;MCM8;FIZ1;PRPF38A;EED;PKMYT1;AP4M1;KIAA0101;ARHGAP11A;CDK1;CDC6;CDC25A
V$E2F1_Q4_01	DDX17;SMC2;YWHAQ;TOPBP1;PAQR4;NDUFA11;ZNF565;CTNND2;DAXX;SASS6;DNMT1;E2F1;THAP8;EZH2;FANCG;DOLK;RALY;PPRC1;POLA2;INTS7;FBXO5;EIF3K;WDR62;ATAD2;H2AFZ;HMGN2;HMGA1;HNRNPA1;HNRNPD;HOXA9;HOXC10;RBPJ;IMPDH2;STMN1;MAT2A;MAZ;MCM2;MCM3;MCM4;MCM6;MCM7;NASP;NCL;GMNN;PCNA;TRMT6;ATP5G2;POLA1;POLE2;ANKHD1;PRKDC;OTUD7B;PTMA;ZNF687;RAD51;RANBP1;RPS19;GINS3;BMP7;SUV39H1;KLF5;UFD1L;USP1;DCTPP1;E2F8;CDC45;MXD3;CASP2;CDCA7;UXT;ACBD6;MCM8;HIST1H2AH;PKMYT1;AP4M1;NUP155;KIAA0101;ARHGAP11A;CDK1;MELK;CDC6;CDC25A

Abbreviations: LeadingEdgeNum, the number of leading edge genes; FDR, false discovery rate from Benjamini and Hochberg from gene set enrichment analysis (GSEA). V$, the annotation found in Molecular Signatures Database (MSigDB) for transcription factors (TF).
